# The Results of Flight upon Normal Health

**Published:** 1914-04-11

**Authors:** 


### THE MEDICAL ASPECTS OF AVIATION.

### The Results of Flight upon Normal Health.

Whenever a new and arduous kind of sport is discovered and obtains a vogue, before very long its influence upon the health of those who pursue it is brought into prominence. Lawn-tennis elbow and cyclist's kyphosis are instances in point; and just lately a discussion has been carried on in the lay press concerning the results of riding astride upon the pelvic organs of the feminine sex.

So, too, it is with flying. The numerous accidents, many of them fatal, which have attended man's rapid conquest of the air this last five years, have been distinguished by a great variety of fractures and other serious injuries; but these hardly deserve any particularisation, since they are the effects of impact with the ground rather than of flying itself. In a new book on aviation* just published some of the medical aspects of aviation are considered in a special chapter written by a medical man.

### Flight as a Remedy.

Before briefly explaining the conclusions set forth as to the results of flight upon a man in good health, it is of interest to recall that aviation has been recommended as a remedy for certain cases of illness, or, at least, of indifferent health. Thus neuralgia is said to disappear like magic in some cases by an ascent in an aeroplane, a statement by no means incredible when it is remembered that neuralgia is often essentially a subjective phenomenon for which no organic basis is discoverable. It is on record also, according to Mr. Hamel, that a well-known balloonist persisted in making an ascent once while suffering from severe influenza, despite the protests of his friends, and that he returned to earth in a few hours entirely cured of his fever. It would, of course, hardly do to allow an influenzal patient to manage an aeroplane, which requires a concentration of faculties, mental and physical, only to be found in the perfectly healthy; but to be a passenger in an aeroplane is a less formidable tax upon the body, and might be permissible. According to these acknowledged experts in aviation, a journey in the air has a very tonic and exhilarating effect, especially when the ascent is made out of the impure air of a large city. Harley Street, say Messrs. Hamel and Turner, to preserve its reputation as the home of all that is best and latest in medical science, will have to put aeroplane flights into its list of up-to-date remedies.

### Air Sickness.

Turning now to the pathological rather than to the therapeutical effects of aviation, *air-sickness* is of importance. Mountain sickness and balloon sickness are, of course, well-known disorders, though their scientific explanation is not settled beyond dispute. Air-sickness is, in essentials, the same disorder, though it appears at a lower altitude than mountain sickness. This is what might be expected, since the aviator ascends so much more rapidly than the mountaineer. Curiously enough, air sickness seems to affect aviators at lower levels than when balloon ascents are made. The leading features of air-sickness are stated to be giddiness, headache, and somnolence, the latter supervening after landing, and sometimes after a considerable interval. Actual nausea is seldom troublesome.

Mr. Adler, who is the author of a chapter on this subject, attributes air-sickness to want of oxygen in the rarified atmosphere of the upper air. The remedy is therefore simple, if this hypothesis is correct—namely, to carry oxygen cylinders and to inhale the gas when very high flying is being attempted. He asserts that pure oxygen is superior to a mixture of oxygen and carbonic acid, which some recommend on the assumption that deprivation of carbonic acid (acapnia) is the true cause of air-sickness.

### Diseases of Airmen.

Beyond this curious result of rapid change in the altitude there are various uncomfortable phenomena to which airmen are liable. The intense cold of flying at high altitudes is naturally one of these detrimental symptoms; it numbs the mental as well as the physical faculties, and is a probable cause of some of the fatal accidents that have occurred. Conjunctivitis is quite frequent, but can be minimised by suitable goggles. Epistaxis occasionally occurs; and on a few occasions blood is said to have poured from the lips, and even from under the nails. Cyanosis of the extremities is quite frequent. Buzzing in the ears is another common inconvenience.

### Women as Aviators.

Great stress is rightly laid upon the prime necessity of keeping absolutely fit physically, and upon the folly of making an ascent if there is the slightest symptom of ill-health or even of temporary mild indisposition. The importance of avoiding tobacco and alcohol is also noted. Aviation is no business for weaklings, and no one should go in for it who has not been passed as thoroughly well equipped constitutionally. We cannot quite follow Mr. Adler in his contention that the family history of the would-be airman should be investigated for cancer, tuberculosis, and apoplexy: that seems an excess of zeal without any real advantage gained. But, at least, it is certain that flying makes demands upon the body and brain which only a man in first-class health and of sound constitution can supply; and it is noteworthy also that Mrs. Maurice Hewlett, one of the few experienced woman aviators, holds that numbers of her sex are not really fitted for flying, and cannot ever expect to compete on even terms with men in that sport.

* *Flying.* By Gustav Hamel and C. C. Turner. Price 12s. 6d. net. Longmans, Green and Co.

